# Enhancement of anti-tumor efficacy of immune checkpoint blockade by alpha-TEA

**DOI:** 10.3389/fimmu.2023.1057702

**Published:** 2023-02-22

**Authors:** William L. Redmond, Melissa J. Kasiewicz, Emmanuel T. Akporiaye

**Affiliations:** ^1^ Earle A. Chiles Research Institute, Providence Cancer Institute, Portland, OR, United States; ^2^ Veana Therapeutics, Portland, OR, United States

**Keywords:** immune checkpoint blockade, PD-1, PD-L1, T cells, alpha-TEA

## Abstract

Cancer immunotherapy such as anti-PD-1/anti-PD-L1 immune checkpoint blockade (ICB) can provide significant clinical benefit in patients with advanced malignancies. However, most patients eventually develop progressive disease, thus necessitating additional therapeutic options. We have developed a novel agent, a-TEA-LS, that selectively induces tumor cell death while sparing healthy tissues, leading to increased activation of tumor-reactive T cells and tumor regression. In the current study, we explored the impact of combined a-TEA-LS + ICB in orthotopic and spontaneously arising murine models of mammary carcinoma. We found that a-TEA-LS + ICB led to increased production of pro-inflammatory cytokines that were associated with a reduction in tumor growth and prolonged survival. Together, these data demonstrate the potential utility of a-TEA-LS + ICB for the treatment of breast cancer and provide the rationale for clinical translation of this novel approach.

## Introduction

Immune checkpoint blockade (ICB) antibodies block the interactions between Programmed Death-1 (PD-1) expressed on activated and “exhausted” T cells and the PD-L1 ligand expressed on cancer cells and tumor-associated immune cells in the tumor microenvironment (TME) to unleash a T cell immune attack against cancer cells (1–3). ICB-targeted therapies have improved clinical outcomes in numerous clinical trials, and several are now approved for multiple cancer indications usually in combination with chemotherapy in the neoadjuvant and adjuvant settings (4–7). While much success has been achieved using these agents, curative responses occur in only a fraction of patients.

Although ICB has made a difference in several cancer indications including melanoma, non-small cell lung cancer, Merkel cell carcinoma, head and neck cancer, and renal cell cancer amongst others, triple negative breast cancer (TNBC) has remained a clinical challenge until the recent FDA approval of pembrolizumab, an anti-PD-1 monoclonal antibody in combination with chemotherapy in early and late stage TNBC (7). Adjuvant chemotherapy, which frequently consists of DNA targeting agents, while necessary, can contribute additional acute toxicity and side effects to the immune-related toxicities inherent in the use of immune checkpoint inhibitors (8–14). These limitations can negatively impact the quality of life of patients during and after treatment and highlight the need for safer, less toxic anti-cancer agents that can be used in combination with ICB to improve patient outcomes.

Alpha-tocopheryloxyacetic acid lysine salt (a-TEA-LS) is a clinical grade small molecule salt form of a-TEA that exhibits tumor cytotoxicity by preferentially targeting dysregulated tumor cell mitochondria to generate toxic reactive oxygen species that trigger apoptotic and autophagic cell death (15–20). This activity concomitantly stimulates the release of “danger signals” including heat shock proteins (HSPs), ATP, calreticulin, and HMGB-1 and generates antigen-containing autophagosomes which stimulate cross-presentation within dendritic cells leading to antigen-specific T cell priming (15, 19, 21).

In pre-clinical proof-of-concept *in vivo* studies, a-TEA increased the frequency of activated CD4^+^ and CD8^+^ T cells in the TME. Furthermore, *in vivo* depletion of CD4^+^ and CD8^+^ T cells in immune competent mice reduced overall survival of a-TEA-treated, tumor-bearing animals implicating T cells in the anti-tumor immune response (21). With a view to utilizing a-TEA as an effective adjuvant to improve the efficacy of ICB and restore anti-tumor activity, we evaluated the anti-tumor activity of a-TEA-LS, a scalable form of a-TEA (22) in combination with PD-1/PD-L1 blockade in three murine models of mammary carcinoma. We report here that a-TEA-LS significantly enhanced the anti-tumor efficacy of anti-PD-1 and anti-PD-L1 in multiple tumor models including inducing complete tumor regression in some instances. These anti-tumor effects were associated with increased effector T cell function at the tumor site.

## Materials and methods

### Preparation of a-tocopheryloxyacetic acid lysine salt (a-TEA-LS)

Alpha-tocopheryloxyacetic acid lysine salt (a-TEA-LS) was synthesized by Olon Ricerca Biosciences LLC (Concord, OH) using a modification of a previously described procedure (22). Briefly, a-TEA-LS was prepared by reacting alpha-D-tocopherol with ethyl bromoacetate to form the ethyl ether intermediate. The ethyl ether intermediate was then reacted with potassium hydroxide to form a-TEA free acid. The lysine salt was formed by adding aqueous lysine solution to a solution of a-TEA in isopropyl alcohol. The lysine salt with its empirical formula of C_37_H_66_N_2_O_6_ and its molecular weight of 634.93 g/mol is a stable crystalline off-white powder. a-TEA-LS was incorporated into the AIN93G diet by Envigo RMS LLC (Indianapolis, IN) at a concentration of 1g a-TEA per kg chow (0.1%).

### Mice

Six to eight-week-old female wild-type BALB/c mice and MMTV-PyMT transgenic mice (FVB/N-Tg(MMTV-PyVT)634Mul/J) were purchased from Jackson Labs (Bar Harbor, ME). All mice were maintained under specific pathogen-free conditions in the Earle A. Chiles Research Institute vivarium at Providence Portland Medical Center (Portland, OR) or at the Experimental Mouse Shared Resource facility at the University of Arizona Cancer Center (Tucson, AZ). Experimental procedures were performed according to the National Institutes of Health Guide for the Care and Use of Laboratory Animals and in accordance with the Institutional Animal Care and Use Committees at both institutions.

### Tumor cell lines and cell culture

4T1 mammary carcinoma cells were grown and maintained in complete RMPI (10% FBS, 1 mol/L HEPES, nonessential amino acids, sodium pyruvate (Lonza), and penicillin–streptomycin-glutamine (Invitrogen)). The Eph4 1424 mouse breast cancer cell line (ATCC #CRL-3071) was grown in DMEM (1X) media (Corning) supplemented with 10% FBS (Omega Scientific). Both tumor cell lines were maintained in 5% CO_2_-95% air humidified atmosphere at 37oC. Cell lines were tested and screened negative for Mycoplasma using the MycoAlert test (Lonza, Walkersville MD).

### *In vivo* tumor studies

BALB/c mice were inoculated with 5 x 10^4^ 4T1 tumor cells subcutaneously (SC) into the right mammary fat pad. Eph4 1424 tumor cells (5 x 10^4^) were injected with Matrigel (Becton Dickinson) into a cleared L4 mammary fat pad (MFP) of BALB/c mice in a total volume of 50 µl. The mice received normal diet until tumor establishment (day 10 post tumor-implantation; ~25 mm^2^) and were then switched to a-TEA-LS-containing mouse chow. Tumor growth was monitored twice weekly by measuring the tumor length (L) and width (W) using calipers and calculating the tumor area as: A = (L × W). Animals were euthanized when tumor area reached >175 mm^2^ (4T1) or at the end of the experiment on Day 84 (Eph4 1424). For the spontaneous tumor model, MMTV-PyMT transgenic mice received normal diet until 6 weeks of age (day 42) and were then switched to a-TEA-LS chow. Control mice remained on matched control diet throughout the study. Mice were inspected for spontaneous tumor growth in the mammary fat pads beginning at week 6. As tumors presented, calipers were used to measure length (L) of the tumor’s longest axis by perpendicular width (W) to calculate tumor area (L x W = A). Individual tumor area was collected for each tumor of each mouse twice weekly. The sum of tumor burden was calculated at each measurement. Mice were euthanized when the total tumor burden was >300 mm^2^.

### Antibody administration

Tumor-bearing mice were treated *via* intraperitoneal (IP) injection in volumes no greater than 200 µL per injection. Three doses of anti-PD-1 (clone RMP1; BioXcell), anti-PD-L1 (clone B7-H1, BioXcell), or control Ab (rat IgG; Sigma) (all at 10 mg/kg) were given every other day when 4T1 tumors reached ~40-60mm^2^ (day 7-10 post-implantation). Eph4 1424 tumor-bearing mice were dosed with anti-PD-L1 or control Ab (200 µg/mouse) on day 10 post-tumor injection when average tumor volume was ~55mm^3^ (range 31-89.85 mm^3^). The mice were dosed every other day for a total of 6 injections. MMTV-PyMT tumor-bearing mice received three doses of anti-PD-L1 or control Ab (10 mg/kg) when they reached 6 weeks of age (day 42, 44 and 46). All mAbs were verified to be endotoxin-free and were injected IP into recipient mice.

### Flow cytometry analysis

Cells were stained for 30 min at 4°C with: CD4 (RM4-5) BV650, CD8 (53–6.7) BV785, CD45 (30-F11) BV570, Fixable Viability Dye eFluor 780, IFN-γ (XMG1.2) PE, IFN-γ (XMG1.2) APC, and Foxp3 (FJK16a) eF450 (ThermoFisher). For intracellular stains, cells were fixed and permeabilized using the Foxp3 Staining Buffer kit (ThermoFisher). Cells were incubated for 30 min at 4^0^ C with: IFN-γ APC and Foxp3 eF450. Cells were collected and analyzed using the LSR II flow cytometer using Diva (BD Biosciences) or FlowJo (Treestar, Ashland, OR) software. For the cytokine bead array, CD4^+^ or CD8^+^ T cells were purified by cell sorting on a FACSAria II Cell Sorter (BD Biosciences) into 1.7 ml tubes containing 400 μl cRPMI. 1x10^6^ cells/well were stimulated with media or plate-bound anti-CD3 and anti-CD28 (5 and 2µg/ml, respectively) in 24-well plates. To measure cytokine production, lymphocytes were incubated in 96-well plates previously coated with anti-CD3 and anti-CD28 mAbs (5 and 2 µg/ml, respectively) in 10% cRPMI and 1.0 μl/ml GolgiPlµg solution (BD Biosciences) for 5h at 37°C. After washing, cells were stained and analyzed by flow cytometry.

### Lymphocyte and TIL isolation

Tumor-infiltrating lymphocytes (TIL) were harvested by cutting tumors into small fragments followed by digestion in 1 mg/ml collagenase and 20 mg/ml DNase (Sigma Aldrich) in RPMI 1640 for 45 min at room temperature. TILs were filtered through 70 µm nylon mesh, then stained for analysis by flow cytometry as described above. Inguinal, axillary, and brachial lymph nodes were harvested and processed to obtain single-cell suspensions. ACK lysing buffer (Lonza) was used to lyse red blood cells. Cells were rinsed with complete RPMI and stained for flow cytometry analysis.

### Cytokine bead array analysis

Supernatant was collected from sorted CD4^+^ and CD8^+^ T-cells cultured in the presence of anti-CD3 and anti-CD28 (5 and 2 μg/ml, respectively) coated 24-well plate for 24 hours for a CBA using a ProcartaPlex Mouse Cytokine/Chemokine Panel 1A 36-Plex kit (EPX360-26012-901; Invitrogen). Data was acquired on a Luminex 200 (R&D Systems).

### Statistical analysis

Statistical significance was determined by unpaired Student t-test (for comparison between two groups), one-way ANOVA for (comparison among three or more groups), or Kaplan-Meier survival (for tumor survival studies) using GraphPad Prism software (GraphPad, San Diego, CA); a *p*-value of <0.05 was considered significant.

## Results

**Figure 1:**
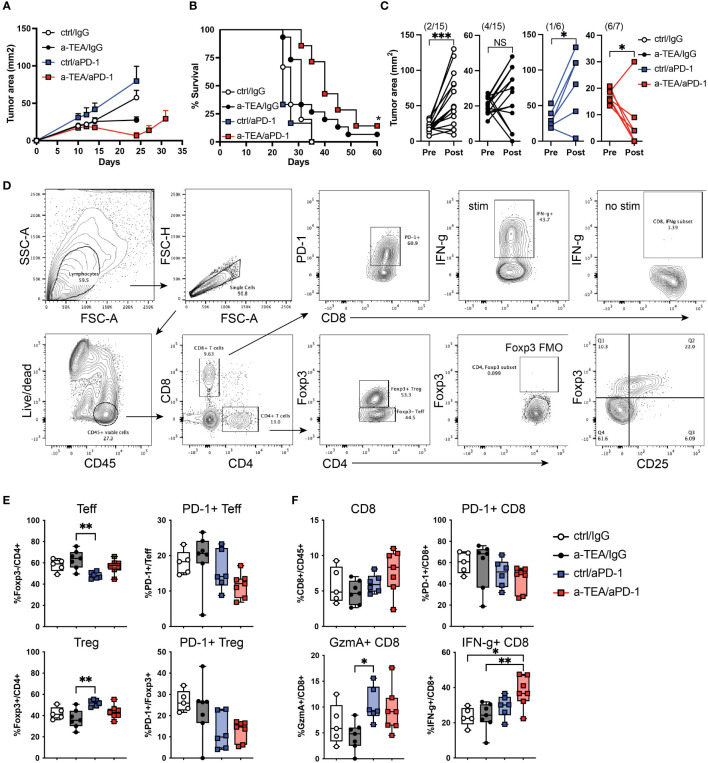
Increased tumor regression and improved survival following a-TEA-LS plus aPD-1 ICB in mice with mammary carcinoma. **(A-C)** 4T1 mammary carcinoma tumor-bearing mice were treated with control diet, IgG (ctrl Ab), a-TEA-LS, and/or aPD-1 and then **(A)** tumor growth (mean+SD) and **(B)** survival were determined. *P<0.05 by Log-rank test. **(C)** Maximum change in tumor size was determined pre- and post-treatment in the indicated treatment groups. Each dot represents an individual mouse. The total number of mice per cohort exhibiting a reduction in tumor size (post-treatment) is depicted in parentheses. *P<0.05, ***P<0.001 by paired t test. **(D-F)** Flow cytometry analysis of tumors from 4T1 tumor-bearing mice. **(D)** Representative flow cytometry graphs from 4T1 tumor-bearing mice treated as in Figure 1A. Seven days after the start of treatment, tumors were harvested for analysis by flow cytometry. **(E, F)** Graphs depicts the frequency of Foxp3^-^CD4^+^ Teff, Foxp3^+^CD4^+^ Treg, and CD8^+^ T cells within the tumor and the extent of PD-1 and IFN-γ expression (mean and range of n=7 mice/group). *P<0.05, **P<0.01 by 1-way ANOVA. NS, not significant.

We investigated the extent to which a-TEA-LS treatment would augment the efficacy of ICB in mammary carcinoma tumor-bearing mice. 4T1 tumor cells, a model of triple-negative breast cancer, were injected orthotopically into wild-type female BALB/c mice and then treated with control diet or a-TEA-LS plus IgG (ctrl) or aPD-1 monoclonal antibody (mAb) (**Figure S1A**). While monotherapy with a-TEA or aPD-1 had a minimal impact on tumor growth or survival (**Figures 1A, B**, respectively), combined a-TEA-LS + aPD-1 therapy led to decreased tumor size (**Figure 1A**) and significantly improved survival (**Figure 1B**). When comparing individual tumor sizes (change in tumor area) between day 10 and 23 post-injection, we observed a transient reduction in tumor size in the majority (6/7; 86%) of a-TEA-LS + aPD-1 treated mice as compared to only 2/15 (13%), 4/15 (27%), and 1/6 (17%) of IgG, a-TEA-LS, and a-PD-1-treated control cohorts, respectively (**Figure 1C**).

Next, we investigated the extent to which a-TEA-LS + ICB affected the frequency and function of tumor infiltrating lymphocytes. 4T1 tumor-bearing mice were treated with a-TEA-Lys in the presence or absence of aPD-1 and then 7 days post-treatment, tumors were harvested and CD4^+^ and CD8^+^ T cell responses were evaluated by flow cytometry (**Figures 1D-F**). Combination a-TEA-LS + ICB therapy had minimal impact on the frequency of effector Foxp3^-^CD4^+^ (Teff) or regulatory Foxp3^+^CD4^+^ (Treg) T cells, although we detected an increase in Tregs following aPD-1 as compared to the a-TEA-LS-treated group. There was also a trend (though not statistically significant among the groups) towards decreased PD-1 expression on CD4^+^ Teff and Tregs receiving combination therapy (**Figure 1E**, right panels).

Analysis of the CD8^+^ T cell compartment revealed no change in cell frequencies following mono- or combination therapy (**Figure 1F**, top left), but a similar trend towards decreased PD-1 expression as was observed in the CD4^+^ T cell compartment (**Figure 1F**, top right). Anti-PD-1 and a-TEA-Lys + anti-PD-1 increased granzyme A expression, although only aPD-1 showed a statistical difference compared to a-TEA-Lys alone (**Figure 1F**, bottom left). However, a-TEA-LS + aPD-1 combination therapy elicited an increased proportion of effector IFN-γ^+^CD8^+^ T cells (**Figure 1F**, bottom right) as compared to ctrl/IgG and a-TEA-LS/IgG-treated controls. These data demonstrate that increased IFN-γ^+^CD8^+^ T cells, rather than the extent of granzyme B expression, is associated with the improved survival observed following a-TEA-LS + aPD-1 therapy.

We also investigated the impact of a-TEA-LS + aPD-1 therapy on the frequency and functional status of myeloid cells including CD11b^+^Ly6C^hi^Ly6G^-^ monocytic myeloid-derived suppressor cells (Mo-MDSC), CD11b^+^Ly6C^med^Ly6G^+^ polymorphonuclear MDSCs (PMN-MDSC), and CD11b^+^F4/80^+^Ly6C^-^Ly6G^-^ macrophages (**Figure S2A**). We did not observe any significant changes in the frequency of these myeloid cell subsets post-treatment (**Figures S2B-D**). There was an increase in arginase within the Mo-MDSC population following a-TEA-LS monotherapy as compared to all other groups (**Figure S2B**) and reduced iNOS following a-TEA-LS as compared to the control group in the PMN-MDSC group (**Figure S2C**), but no differences were observed in macrophages across treatments (**Figure S2D**).

**Figure 2:**
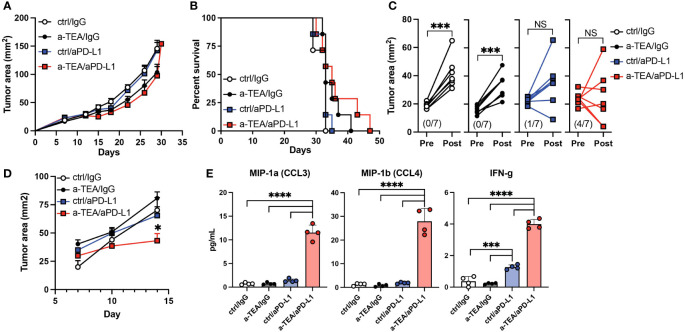
Impact of combined a-TEA-LS plus aPD-L1 ICB on tumor growth and T cell differentiation. **(A-E)** 4T1 mammary carcinoma tumor-bearing mice were treated with control diet, IgG (ctrl Ab), a-TEA-LS, and/or aPD-L1 and then **(A)** tumor growth (mean+SD) and **(B)** survival were determined. **(C)** Maximum change in tumor size was determined pre- and post-treatment in the indicated treatment groups. Each dot represents an individual mouse. The total number of mice per cohort exhibiting a reduction in tumor size (post-treatment) is depicted in parentheses. ***P<0.001 by paired t test. **(D)** Tumor growth of 4T1 tumor-bearing mice treated as described above and then **(E)** CD8^+^ T cells were isolated from the lymph nodes 8 days following treatment. Cells were restimulated with aCD3 for 24 hours *in vitro* and then supernatants were collected and protein expression determined by multiplex ELISA. Each dot represents an individual mouse. Graphs represent the mean and range from 4 mice per group from 1 of 2 independent experiments. *P<0.05, ***P<0.001, ****P<0.0001 by 1-way ANOVA. NS, not significant.

Recent analyses have suggested differences in the therapeutic efficacy of aPD-1 vs. aPD-L1 for the treatment of TNBC (23–26). It is also known that the preclinical aPD-1 and aPD-L1 mAbs have differential binding affinity to their targets, which may impact their efficacy (27). Therefore, we sought to evaluate the efficacy of a-TEA-LS + aPD-L1 therapy in 4T1 tumor-bearing mice. Combined a-TEA-LS + aPD-L1 therapy was associated with a slight decrease in tumor size (**Figure 2A**) and some improved survival (**Figure 2B**), though these did not reach statistical significance. Our analysis of changes in individual tumor area revealed a reduction in tumor size in the majority (4/7; 57%) of a-TEA-LS + aPD-L1-treated mice as compared to only 0/7 (0%), 0/7 (0%), and 1/7 (14%) of IgG, a-TEA-LS, and a-PD-L1-treated control cohorts, respectively (**Figure 2C**). In another cohort of mice, we observed a significant reduction in tumor size in the a-TEA-LS + a-PD-L1 combination group at an earlier time of tumor harvest as compared to monotherapy-treated controls (**Figure 2D**) along with a significant increase in several effector proteins including MIP-1α/CCL3, MIP-1β/CCL4, and IFN-γ following a-TEA-LS + aPD-L1 (**Figure 2E**). Global analysis of the cytokine milieu after a-TEA-LS + ICB therapy suggests that this combination also drives an anti-tumor proinflammatory Th1-polarized profile to support tumor control as no significant changes were observed in Th2 cytokines (SF3).

**Figure 3:**
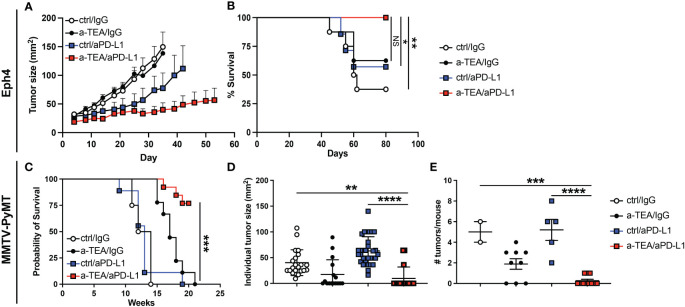
Combined a-TEA-LS plus aPD-L1 ICB reduces tumor burden and extends survival in Eph4 1424 tumor-bearing mice and in the PyMT-MMTV Tg model of spontaneously arising mammary carcinoma. **(A, B)** Eph4 1424 mammary carcinoma tumor-bearing mice were treated with control diet, IgG (ctrl Ab), a-TEA-LS, and/or aPD-L1 and then **(A)** tumor growth (mean+SD) and **(B)** survival were determined (n=8/group). C-E) PyMT-MMTV Tg mice were treated with control diet/IgG (n=4), a-TEA-LS/IgG (n=9), ctrl/aPD-L1 (n=8), or a-TEA-LS/aPD-L1 (n=13) starting at 6 weeks of age. **(C)** Survival, **(D)** individual tumor size (left panel), and **(E)** number of tumors per mouse (all at week 13) was determined. Graphs depict the mean+/-SEM. **(B, D)** *P<0.05, **P<0.01, ***P<0.001 by Log-rank test; **(E)** **P<0.01, ***P<0.001, ****P<0.0001 by 1-way ANOVA. NS, not significant.

Given the limited anti-tumor effect observed in 4T1 tumor-bearing mice, we next asked whether combined a-TEA-LS + aPD-L1 therapy exhibited therapeutic efficacy in two additional models, Eph4 1424 tumor-bearing mice, which express constitutively active MEK1 (28), and the PyMT-MMTV Tg model of spontaneously arising mammary carcinoma. Combination a-TEA-LS + aPD-L1 therapy decreased tumor growth and improved survival in Eph4 1424 tumor-bearing mice (**Figures 3A, B**). Next, PyMT-MMTV Tg mice, which express the Polyoma Virus middle T antigen under the control of mammary tumor virus promoter/enhancer, were treated with control diet, IgG, a-TEA-LS, and/or aPD-L1 starting at 6 weeks of age (**Figure S1B**) and then tumor growth and survival were determined. Combined a-TEA-LS + aPD-L1 therapy significantly improved survival (**Figure 3C**), which was associated with a significant reduction in individual tumor size (**Figure 3D**) and number of tumors per mouse (**Figure 3E**) as compared to aPD-L1 alone.

## Discussion

While ICB has provided a new treatment option for patients with advanced malignancies, most patients still develop progressive disease highlighting the critical need for additional therapies. In this study, we investigated the extent to which a novel therapy, a-TEA-LS, in conjunction with aPD-1 or aPD-L1 ICB would impact the growth of murine mammary carcinomas. Our data revealed that combined a-TEA-LS + aPD-1 ICB therapy significantly reduced tumor growth and was associated with the activation of effector CD8^+^ T cells within the TME as compared to controls (**Figure 1**). The increased therapeutic efficacy of a-TEA-LS + aPD-1 therapy was associated with a trend of lower PD-1^+^ CD4^+^ and CD8^+^ T cells (**Figures 1E, F**), suggesting they may be less exhausted or suppressed than control-treated mice. Supporting this hypothesis, we observed a significant increase in effector IFN-γ^+^ CD8^+^ T cells following combination therapy as compared to controls (**Figure 1F**, lower right panel).

In contrast, we observed less potent effects on tumor growth or survival following a-TEA-LS + aPD-L1 treatment in the 4T1 tumor model (**Figures 2A, B**, respectively). The reasons for this differential impact on tumor growth and survival as compared to a-TEA-LS + aPD-1 therapy remain unclear. For example, we observed a significant increase in the proinflammatory cytokine IFN-γ following a-TEA-LS plus aPD-1 or aPD-L1 (**Figures 1F**, **2E**), but this was not sufficient for generating equivalent anti-tumor responses. The reduced efficacy of aPD-L1 may reflect differences in the biochemical properties of murine aPD-1 vs. a-PD-L1 mAbs such as affinity for their targets (27). We are conducting additional studies to explore further the differences in therapeutic efficacy between these agents in murine models of mammary carcinoma.

Given the slightly reduced responsiveness of a-TEA-LS + aPD-L1 ICB in the 4T1 model, we explored the therapeutic efficacy of this combination therapy in two additional tumor models, Eph4 1424 (MEK1-driven) and the PyMT-MMTV Tg model of spontaneously arising mammary carcinoma. Combination therapy led to improved survival in both models (**Figure 3**) and we also observed a significant decrease in tumor foci and total tumor burden in the PyMT-MMTV Tg model following a-TEA-LS + aPD-L1 immunotherapy (**Figures 3D, E**), highlighting the therapeutic impact of this approach.

Recent work has highlighted the important contribution of myeloid cells to immunosuppression with the TME (29–33). However, we did not observe any significant changes in the frequency or functional status of monocytic (Mo-MDSC) or polymorphonuclear myeloid-derived suppressor cells (PMN-MDSC) or tumor-associated macrophages post-treatment (SF2), suggesting that the efficacy of a-TEA-LS + ICB was likely primarily associated with promoting T cell function, rather than reducing MDSC-mediated immune suppression. Similarly, we detected few changes in the Treg compartment in terms of their frequency or phenotype (**Figure 1E** and data not shown), although the impact on Treg (or MDSC)-specific suppression has not been assessed.

Previous studies have demonstrated the ability of a-TEA-LS to promote cross-presentation of tumor-associated antigens by driving increased tumor cell-specific autophagy (19). It is likely that the addition of ICB mitigates the induction of T cell exhaustion following treatment with a-TEA-LS, thus enabling a more potent anti-tumor response to occur. While the current study provides some insight into the mechanisms by which a-TEA-LS + ICB supports tumor regression through enhanced anti-tumor immunity, future work is planned to further understand how this approach alters the TME. For example, we plan to use single-cell RNA-seq + scTCR-seq analysis to fully characterize the entirety of the TME at single-cell resolution as well as using multiplex immunohistochemistry to investigate the impact of combination therapy on the spatial organization of the TME.

Most importantly, these data provide the rationale to explore combined a-TEA-LS + ICB therapy for patients with advanced malignancies, including breast cancer. A recent study evaluated the safety of a-TEA-LS in a phase I clinical trial as a monotherapy in patients with advanced cancer (NCT02192346). Currently, escalating doses of a-TEA-LS in combination with trastuzumab (anti-HER2 mAb) are being evaluated for safety in a Phase Ib trial in patients with treatment-refractory HER2^+^ metastatic breast cancer (NCT04120246). Based on the safety profile of a-TEA-LS and the enhanced anti-tumor efficacy demonstrated by a-TEA-LS + ICB in pre-clinical models of breast cancer, a-TEA-LS has the potential to be used as an adjuvant drug to improve the effectiveness of ICB in human breast cancer.

## Data availability statement

The raw data supporting the conclusions of this article will be made available by the authors, without undue reservation.

## Ethics statement

Experimental procedures were performed according to the National Institutes of Health Guide for the Care and Use of Laboratory Animals and in accordance with the Earle A. Chiles Research Institute (EACRI) Institutional Animal Care and Use Committee (Animal Welfare Assurance No. A3913–01).

## Author contributions

WR made substantial contributions to the conception, design, data analysis and interpretation, and drafted the manuscript. MK made substantial contributions to data acquisition and analysis. EA provided key reagents and contributed to the conception and design of the experiments and drafted the manuscript. All authors contributed to the article and approved the submitted version.

## References

[1] Page DB Postow MA Callahan MK Allison JP Wolchok JD. Immune modulation in cancer with antibodies. Annu Rev Med (2014) 65:185–202. doi: 10.1146/annurev-med-092012-112807 24188664

[2] Emens LA Ascierto PA Darcy PK Demaria S Eggermont AMM Redmond WL. Cancer immunotherapy: Opportunities and challenges in the rapidly evolving clinical landscape. Eur J Cancer (2017) 81:116–29. doi: 10.1016/j.ejca.2017.01.035 28623775

[3] Wei SC Duffy CR Allison JP. Fundamental mechanisms of immune checkpoint blockade therapy. Cancer Discovery (2018) 8:1069–86. doi: 10.1158/2159-8290.CD-18-0367 30115704

[4] Choueiri TK Tomczak P Park SH Venugopal B Ferguson T Chang YH. Adjuvant pembrolizumab after nephrectomy in renal-cell carcinoma. N Engl J Med (2021) 385:683–94. doi: 10.1056/NEJMoa2106391 34407342

[5] Forde PM Spicer J Lu S Provencio M Mitsudomi T Awad MM. Neoadjuvant nivolumab plus chemotherapy in resectable lung cancer. N Engl J Med (2022) 386:1973–85. doi: 10.1056/NEJMoa2202170 PMC9844511 35403841

[6] Luke JJ Rutkowski P Queirolo P Del Vecchio M Mackiewicz J Chiarion-Sileni V. Pembrolizumab versus placebo as adjuvant therapy in completely resected stage IIB or IIC melanoma (KEYNOTE-716): A randomised, double-blind, phase 3 trial. Lancet (2022) 399:1718–29. doi: 10.1016/S0140-6736(22)00562-1 35367007

[7] Schmid P Cortes J Dent R Pusztai L Mcarthur H Kummel S. Event-free survival with pembrolizumab in early triple-negative breast cancer. N Engl J Med (2022) 386:556–67. doi: 10.1056/NEJMoa2112651 35139274

[8] Azim HA Jr. De Azambuja E Colozza M Bines J Piccart MJ. Long-term toxic effects of adjuvant chemotherapy in breast cancer. Ann Oncol (2011) 22:1939–47. doi: 10.1093/annonc/mdq683 21289366

[9] Fujii T Le Du F Xiao L Kogawa T Barcenas CH Alvarez RH. Effectiveness of an adjuvant chemotherapy regimen for early-stage breast cancer: A systematic review and network meta-analysis. JAMA Oncol (2015) 1:1311–8. doi: 10.1001/jamaoncol.2015.3062 PMC5575939 26402167

[10] Cortes J Cescon DW Rugo HS Nowecki Z Im SA Yusof MM. Pembrolizumab plus chemotherapy versus placebo plus chemotherapy for previously untreated locally recurrent inoperable or metastatic triple-negative breast cancer (KEYNOTE-355): A randomised, placebo-controlled, double-blind, phase 3 clinical trial. Lancet (2020) 396:1817–28. doi: 10.1016/S0140-6736(20)32531-9 33278935

[11] Delgado-Ramos GM Nasir SS Wang J Schwartzberg LS. Real-world evaluation of effectiveness and tolerance of chemotherapy for early-stage breast cancer in older women. Breast Cancer Res Treat (2020) 182:247–58. doi: 10.1007/s10549-020-05684-5 32447595

[12] Larroquette M Domblides C Lefort F Lasserre M Quivy A Sionneau B. Combining immune checkpoint inhibitors with chemotherapy in advanced solid tumours: A review. Eur J Cancer (2021) 158:47–62. doi: 10.1016/j.ejca.2021.09.013 34655837

[13] Miles D Gligorov J Andre F Cameron D Schneeweiss A Barrios C. Primary results from IMpassion131, a double-blind, placebo-controlled, randomised phase III trial of first-line paclitaxel with or without atezolizumab for unresectable locally advanced/metastatic triple-negative breast cancer. Ann Oncol (2021) 32:994–1004. doi: 10.1016/j.annonc.2021.05.801 34219000

[14] Xin Y Shen G Zheng Y Guan Y Huo X Li J. Immune checkpoint inhibitors plus neoadjuvant chemotherapy in early triple-negative breast cancer: A systematic review and meta-analysis. BMC Cancer (2021) 21:1261. doi: 10.1186/s12885-021-08997-w 34814874 PMC8609839

[15] Hahn T Szabo L Gold M Ramanathapuram L Hurley LH Akporiaye ET. Dietary administration of the proapoptotic vitamin e analogue alpha-tocopheryloxyacetic acid inhibits metastatic murine breast cancer. Cancer Res (2006) 66:9374–8. doi: 10.1158/0008-5472.CAN-06-2403 17018590

[16] Neuzil J Dong LF Ramanathapuram L Hahn T Chladova M Wang XF. Vitamin e analogues as a novel group of mitocans: Anti-cancer agents that act by targeting mitochondria. Mol Aspects Med (2007) 28:607–45. doi: 10.1016/j.mam.2007.02.003 17499351

[17] Hahn T Fried K Hurley LH Akporiaye ET. Orally active alpha-tocopheryloxyacetic acid suppresses tumor growth and multiplicity of spontaneous murine breast cancer. Mol Cancer Ther (2009) 8:1570–8. doi: 10.1158/1535-7163.MCT-08-1079 PMC3693733 19509249

[18] Dong LF Grant G Massa H Zobalova R Akporiaye E Neuzil J. Alpha-tocopheryloxyacetic acid is superior to alpha-tocopheryl succinate in suppressing HER2-high breast carcinomas due to its higher stability. Int J Cancer (2012) 131:1052–8. doi: 10.1002/ijc.26489 22038845

[19] Li Y Hahn T Garrison K Cui ZH Thorburn A Thorburn J. The vitamin e analogue alpha-TEA stimulates tumor autophagy and enhances antigen cross-presentation. Cancer Res (2012) 72:3535–45. doi: 10.1158/0008-5472.CAN-11-3103 PMC3576035 22745370

[20] Rodriguez-Enriquez S Hernandez-Esquivel L Marin-Hernandez A Dong LF Akporiaye ET Neuzil J. Molecular mechanism for the selective impairment of cancer mitochondrial function by a mitochondrially targeted vitamin e analogue. Biochim Biophys Acta (2012) 1817:1597–607. doi: 10.1016/j.bbabio.2012.05.005 22627082

[21] Hahn T Jagadish B Mash EA Garrison K Akporiaye ET. Alpha-tocopheryloxyacetic acid: A novel chemotherapeutic that stimulates the antitumor immune response. Breast Cancer Res (2011) 13:R4. doi: 10.1186/bcr2808 21232138 PMC3109570

[22] Guerrouahen BS Hahn T Alderman Z Curti B Urba W Akporiaye ET. GMP-grade alpha-TEA lysine salt: A 28-day oral toxicity and toxicokinetic study with a 28-day recovery period in beagle dogs. BMC Cancer (2016) 16:199. doi: 10.1186/s12885-016-2220-6 26957307 PMC4784284

[23] Chen S Zhang Z Zheng X Tao H Zhang S Ma J. Response efficacy of PD-1 and PD-L1 inhibitors in clinical trials: A systematic review and meta-analysis. Front Oncol (2021) 11:562315. doi: 10.3389/fonc.2021.562315 33937012 PMC8085334

[24] Li CJ Lin LT Hou MF Chu PY. PDL1/PD1 blockade in breast cancer: The immunotherapy era (Review). Oncol Rep (2021) 45:5–12. doi: 10.3892/or.2020.7831 33416128

[25] Dixon-Douglas J Loibl S Denkert C Telli M Loi S. Integrating immunotherapy into the treatment landscape for patients with triple-negative breast cancer. Am Soc Clin Oncol Educ Book (2022) 42:1–13. doi: 10.1200/EDBK_351186 35649211

[26] Wang C. A meta-analysis of efficacy and safety of PD-1/PD-L1 inhibitors in triple-negative breast cancer. J Oncol (2022) 2022:2407211. doi: 10.1155/2022/2407211 35096057 PMC8799355

[27] Bu MT Yuan L Klee AN Freeman GJ. A comparison of murine PD-1 and PD-L1 monoclonal antibodies. Monoclon Antib Immunodiagn Immunother (2022) 41:202–9. doi: 10.1089/mab.2021.0068 PMC9451140 35925787

[28] Pinkas J Leder P. MEK1 signaling mediates transformation and metastasis of EpH4 mammary epithelial cells independent of an epithelial to mesenchymal transition. Cancer Res (2002) 62:4781–90. 12183438

[29] Ruffell B Au A Rugo HS Esserman LJ Hwang ES Coussens LM. Leukocyte composition of human breast cancer. Proc Natl Acad Sci U.S.A. (2012) 109:2796–801. doi: 10.1073/pnas.1104303108 PMC3287000 21825174

[30] Coussens LM Zitvogel L Palucka AK. Neutralizing tumor-promoting chronic inflammation: a magic bullet? Science (2013) 339:286–91. doi: 10.1126/science.1232227 PMC3591506 23329041

[31] Markowitz J Wesolowski R Papenfuss T Brooks TR Carson WE 3rd. Myeloid-derived suppressor cells in breast cancer. Breast Cancer Res Treat (2013) 140:13–21. doi: 10.1007/s10549-013-2618-7 23828498 PMC3773691

[32] Alshetaiwi H Pervolarakis N Mcintyre LL Ma D Nguyen Q Rath JA. Defining the emergence of myeloid-derived suppressor cells in breast cancer using single-cell transcriptomics. Sci Immunol (2020) 5. doi: 10.1126/sciimmunol.aay6017 PMC7219211 32086381

[33] Blaye C Boyer T Peyraud F Domblides C Larmonier N. Beyond immunosuppression: The multifaceted functions of tumor-promoting myeloid cells in breast cancers. Front Immunol (2022) 13:838040. doi: 10.3389/fimmu.2022.838040 35309358 PMC8927658

