# Supplementary figures and images for: Enhancement of anti-tumor efficacy of immune checkpoint blockade by alpha-TEA
Source: Front Immunol. 2023 Feb 22;14:1057702. doi: 10.3389/fimmu.2023.1057702 (PMC9992800; doi:10.3389/fimmu.2023.1057702)

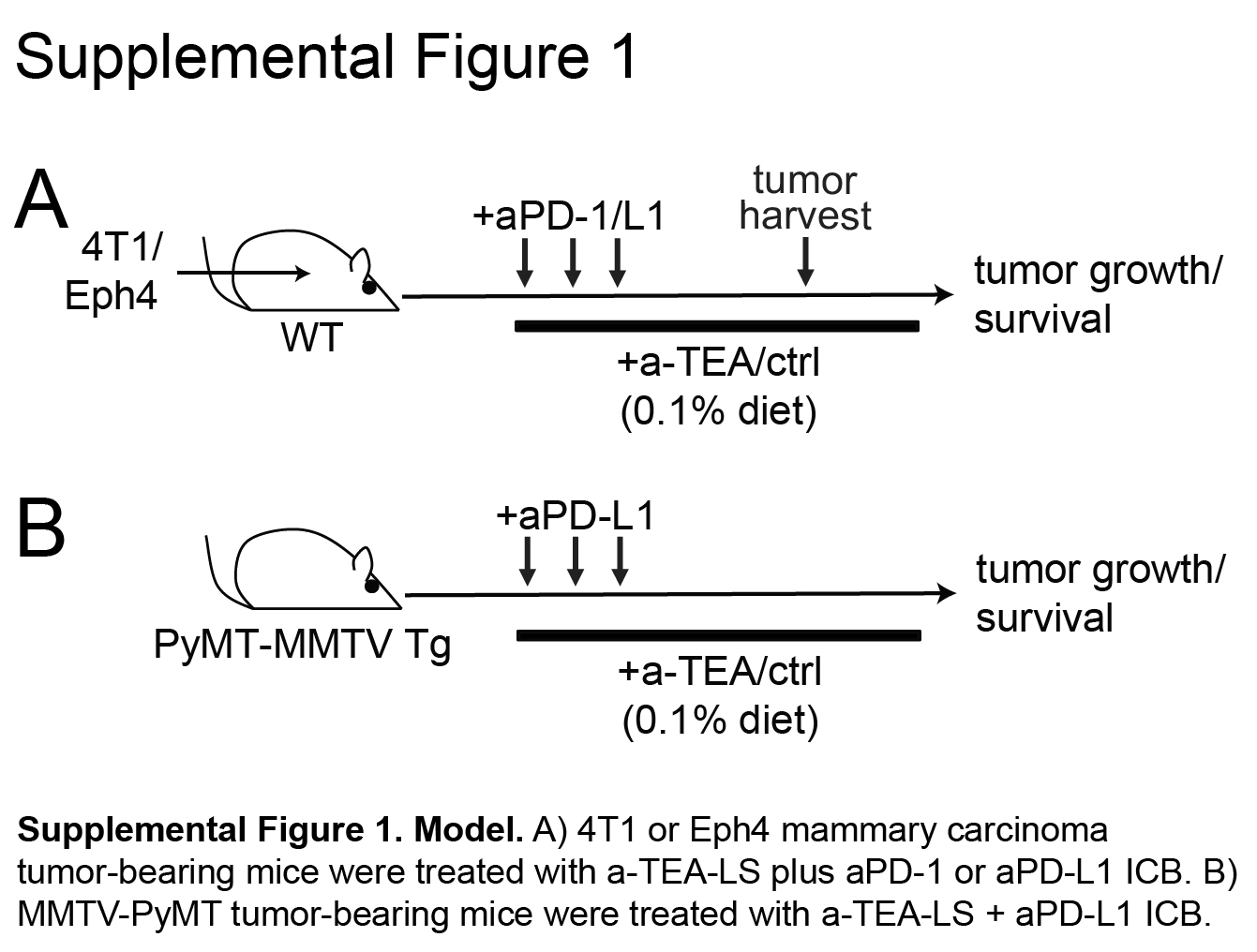

Supplement: Supplementary file 1 [file Image_1.jpg]

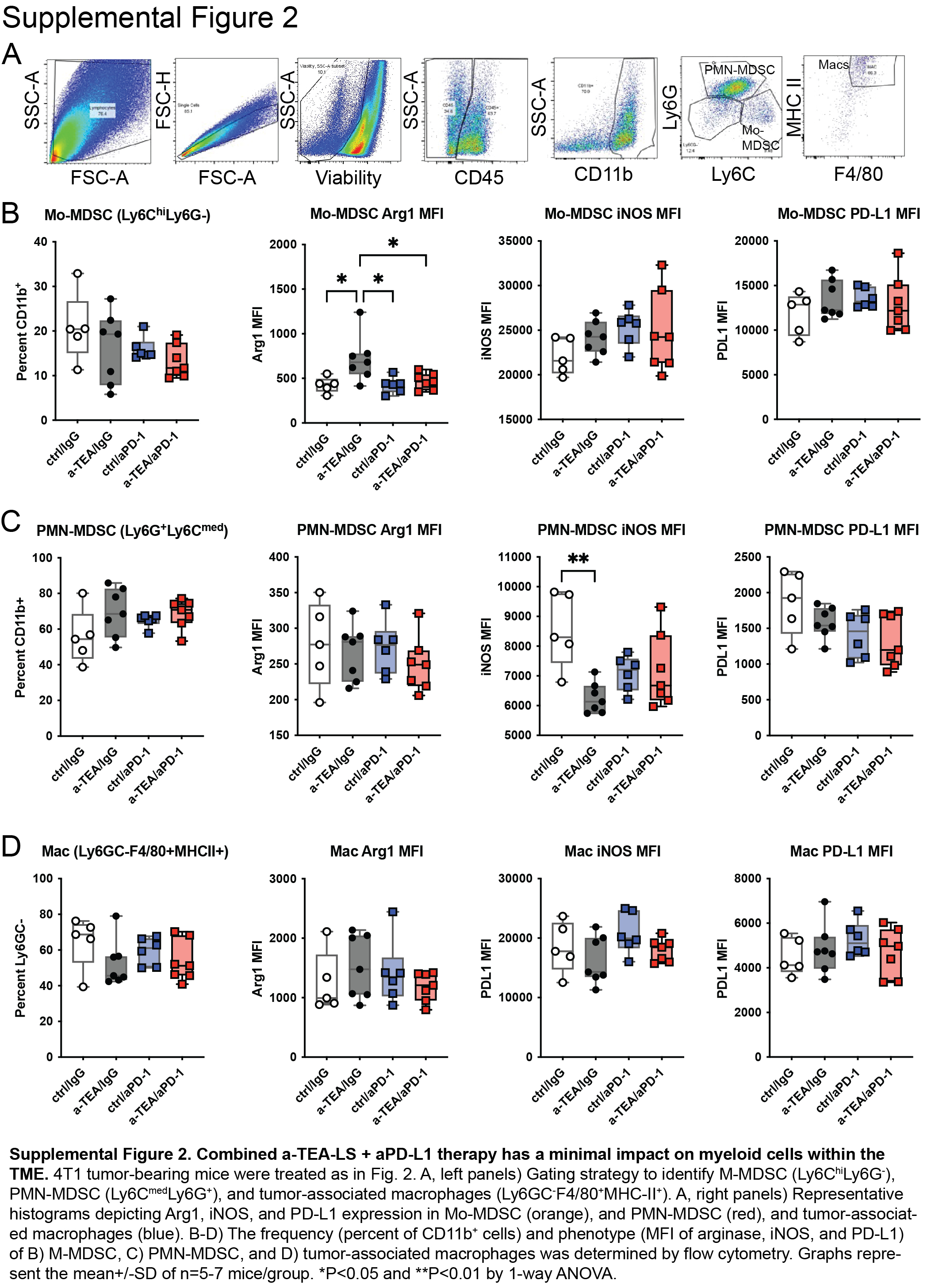

Supplement: Supplementary file 2 [file Image_2.jpg]

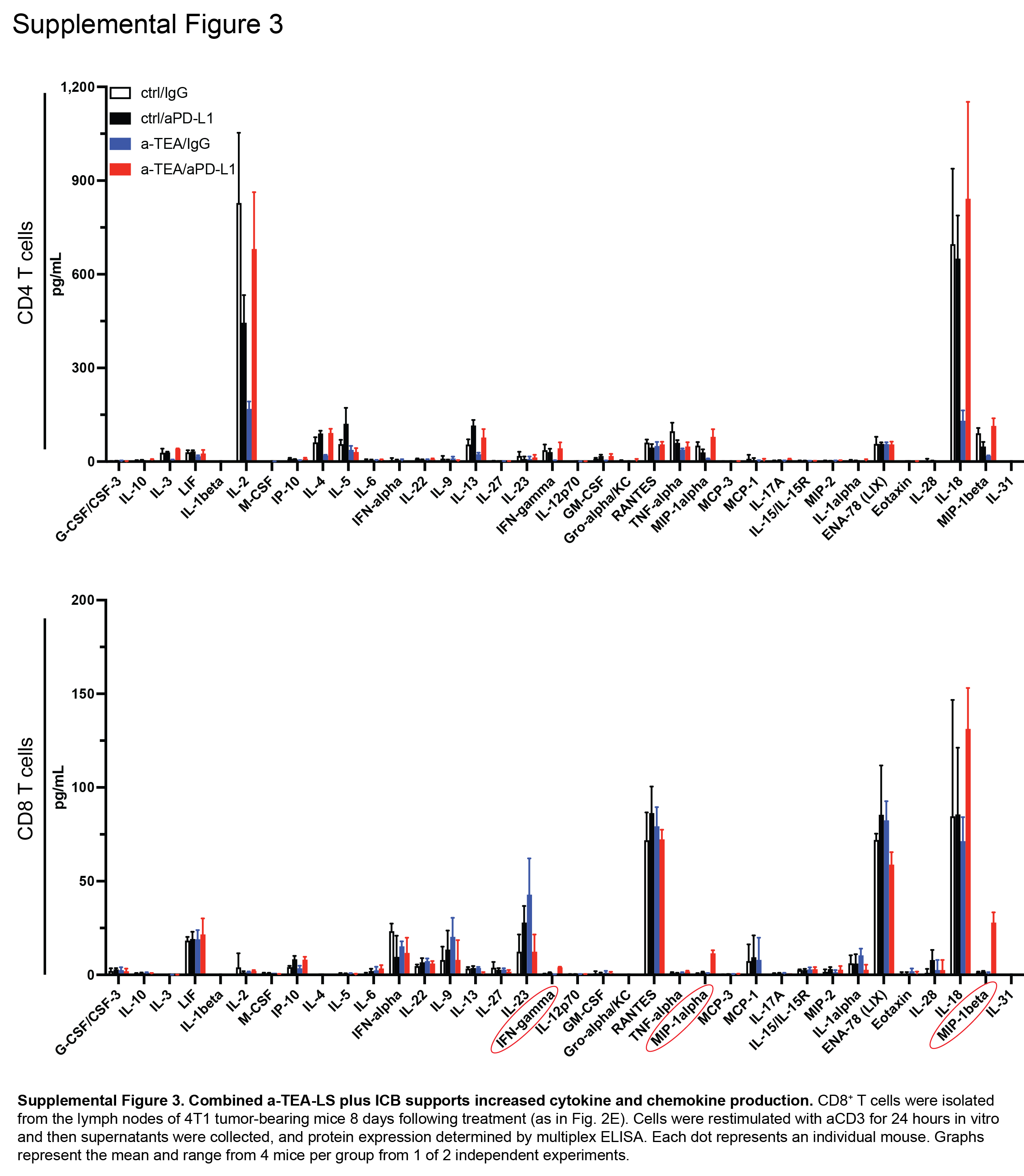

Supplement: Supplementary file 3 [file Image_3.jpg]
